# Using Femtosecond Laser Light-Activated Materials: The Biomimetic Dentin Remineralization Was Monitored by Laser-Induced Breakdown Spectroscopy

**DOI:** 10.3390/medicina59030591

**Published:** 2023-03-16

**Authors:** Howida Kandil, Esraa Ahmed, Nada Fouad, Ola Ali Dabbous, Maha Niazy, Tarek Mohamed

**Affiliations:** 1 Department of Medical Laser Applications, Laser Institute for Research Application, Beni-Suef University, Beni Suef 2722165, Egypt; howida.kandil@gmail.com (H.K.); esr3ahmed@lira.bsu.edu.eg (E.A.); nadafouad@lira.bsu.edu.eg (N.F.); 2 Department of Medical Applications of Lasers, National Institute of Laser Enhanced Science (NILES), Cairo University, Giza 12611, Egypt; oladabous@yahoo.com; 3 Operative Dentistry Department, Faculty of Dental Medicine for Girls, Al-Azhar University, Cairo 4434004, Egypt; drniazy_maha@hotmail.com

**Keywords:** ablation, antimicrobial agents, caries, dentin tissue, enamel, femtosecond, LIBS, tissue stimulation

## Abstract

*Introduction*: The purpose of this study is to investigate and compare the effects of the antimicrobial agents *Moringa oleifera* and bioactive glass nanoparticles activated by femtosecond laser light on the biomimetic dentin remineralization using teeth having carious dentin ICDAS code 3. *Methods and Materials*: A total of 27 dentin surface samples were divided into three groups: the first group was treated with a *Moringa oleifera* extract, while the second group was treated with bioactive glass nanoparticles, and as for the control group, the third group received no additional agent. All groups were subjected to femtosecond laser light at three different wavelengths: 390 nm, 445 nm, and 780 nm. The photoactivation of each sample was achieved using the femtosecond laser light for 5 min with an average power rating of 300 mW, a pulse duration of 100 fs, and a pulse repetition rate of 80 Hz. The mineral content of the samples was obtained and analyzed using the laser-induced breakdown spectroscopy (LIBS). The LIBS analysis was conducted with the following laser light parameters: average power of ~215 mW, wavelength of 532 nm, pulse duration of 10 ns, and a pulse repetition rate of 10 Hz. *Results*: Most studied samples exhibited a relative increase in the mineral content that may enhance biomimetic remineralization. *Moringa oleifera* photoactivated by femtosecond laser light at 445 nm achieved a significant increase in mineral content. *Conclusion*: Using the femtosecond laser light to activate the relatively cheap and commercially available antimicrobial agent *Moringa oleifera* supports the strategy of minimal invasive approaches for the treatment and biomimetic remineralization of carious dentin ICDAS code 3.

## 1. Introduction

In the modern age of dentistry, treating dental caries is expensive. In the United States alone, it costs USD 100 million annually as it necessitates the use of dental materials, which have some drawbacks [1], such as polymerization shrinkage, microleakage, tooth fracture, and recurrent caries. Other factors, such as those related to the patient, include lack of follow-up, improper dental hygiene, tooth morphology, and the patient’s age.

Recent dental treatment approaches aim to be more conservative by minimizing drilling in the intact tooth structure. Biomimetic remineralization is one of the best techniques that merely eliminate the unhealthy dental tissue, then induce the mineral content to mimic the healthy tissue [2]. Normally, dental tissue exhibits both demineralization and remineralization; however, when dental caries is present, the process of remineralization is interrupted, and the demineralization outweighs the remineralization [3]. The biomimetic technique, which utilizes a natural or synthetic agent to simulate, regenerate, and repair the damaged tooth structure of the caries-infected dental tissue, is one of the ideal conservative alternative treatments.

Most of the tooth composition is the dentin layer, which is a hard, porous layer of tissue with a yellow light appearance [4]. Dentin tissue can remineralize and regenerate and it is situated beneath the tooths external layer [5]. Therefore, the remineralization of dentin that have been impacted by caries is one of the clinical instances where this biomimetic remineralization process has good potential.

The minerals that form the dental tissue are termed as hydroxyapatite crystals, which are composed of calcium (Ca), phosphorous (P), and oxygen (O) elements. In addition to these three elements, dental tissue also contains significant amounts of carbon (C), magnesium (Mg), sodium (Na), fluoride (F), and other trace elements [6]. To track this elemental composition throughout the study, the laser-induced breakdown spectroscopy (LIBS) is used, which permits the analysis of elemental composition changes qualitatively and quantitively. The elemental analysis in LIBS, a non-contact technique, is based on the spectral analysis of plasma emission produced by exposure to high-power laser pulses [7]. Since it is rapid and sensitive, it is not disruptive and can assess extremely minute volumes in situ in real time [7]. As a result, LIBS also permits the evaluation of the remineralization of dentin. LIBS is less expensive than alternative techniques for elemental analysis, such as inductively coupled plasma-atomic emission spectroscopy.

Nowadays, laser devices are becoming more common in all branches of dentistry. Due to its ultra-short laser pulses, the femtosecond laser is regarded in the realm of dentistry as a minimally invasive treatment since it ablates cavities without creating a crack [8]. For instance, a novel method using the Quanta-Ray femtosecond laser Inspire HF100 has been shown to be bactericidal against a variety of bacteria, including drug-resistant species, by removing the bacterial biofilm that causes dental disease [9]. However, the therapeutic effectiveness of femtosecond laser light on dentin remineralization is currently not sufficiently supported by research. To the knowledge of the authors, no prospective research comparing femtosecond laser light dental treatments with conventional techniques has been published. Therefore, more scientific evidence is required on the application of femtosecond laser light to justify its usage, as clinicians start exploring this new therapy and determining how to apply it in clinical practice.

The biomimetic remineralization of dentin has been successfully accomplished by several natural and synthetic antibacterial agents. For instance, the medicinal plant *Moringa oleifera* has proven to be a highly effective source of antibacterial compounds. It has been demonstrated that *Moringa oleifera* works well for remineralizing enamel and dentin [10]. The application of *Moringa oleifera* resulted in a significant improvement in the calcium and phosphorus levels, and as such, remineralization occurred in the casein phosphopeptide–amorphous calcium phosphate fluoride (CPP-ACPF) groups. Another promising antimicrobial agent is bioactive glass, which is useful for remineralizing dentin because it can cause the development of apatite in the dentin.

Several studies have shown the value of *Moringa oleifera* in different applications [11]. For example, the minerals found in *Moringa oleifera* include most macro-minerals in high concentrations, with calcium being a relevant one [12]. This suggests that it may be utilized to support the development and repair of teeth and bones. It has also been shown that different components of the *Moringa oleifera* plant have therapeutic qualities. For instance, the roots are analgesic, anti-inflammatory, anticarcinogenic, antidiabetic, and antihypertensive, whereas the leaves are antiproliferative and diuretic. The flower part is antipsychotic, and the seeds are antidiuretic and anti-asthmatic [13]. Other than being used as an herbal remedy, recently, it has also been investigated in fields unrelated to medicine, such as ground water purification [14].

Bioactive compounds have also shown their ability in antimicrobial applications [15]. For example, bioactive glass nanoparticles are also employed for biomimetic remineralization as when applied, it creates a biological link to the tissue, which in turn may cause a biological reaction with the tissue [16]. Enamel and dentin, as well as bone, are mostly made of mineralized hard tissue called hydroxyapatite, a crystalline calcium phosphate, Ca_10_(PO_4_)_6_(OH)_2_. It has been shown that bioactive glass causes dentin remineralization as it causes the development of apatite in the dentin [17,18].

The effects of activating antimicrobial compounds with femtosecond laser light on dentin remain to be addressed. The ultraviolet, visible, and near-infrared wavelengths of the electromagnetic spectrum are of specific interest as at these wavelengths, femtosecond laser applications have demonstrated strong bactericidal efficiency on major infectious bacterial pathogens. This may be accomplished by the contact-active strategy, which involves immobilizing antimicrobial compounds on an infected surface and activating them with a laser to render them lethal to bacteria upon surface contact and reduce the number of antimicrobial agents that are significantly leached from the functionalized surface [19].

The purpose of this research is to examine and compare the biomimetic remineralization effects of dentin when applying the antibacterial agents *Moringa oleifera* and bioactive glass nanoparticles activated by femtosecond laser light on teeth with carious dentin ICDAS code 3. The evaluation analysis is conducted by the LIBS method.

## 2. Materials and Methods

### 2.1. Materials

**Figure 1:**
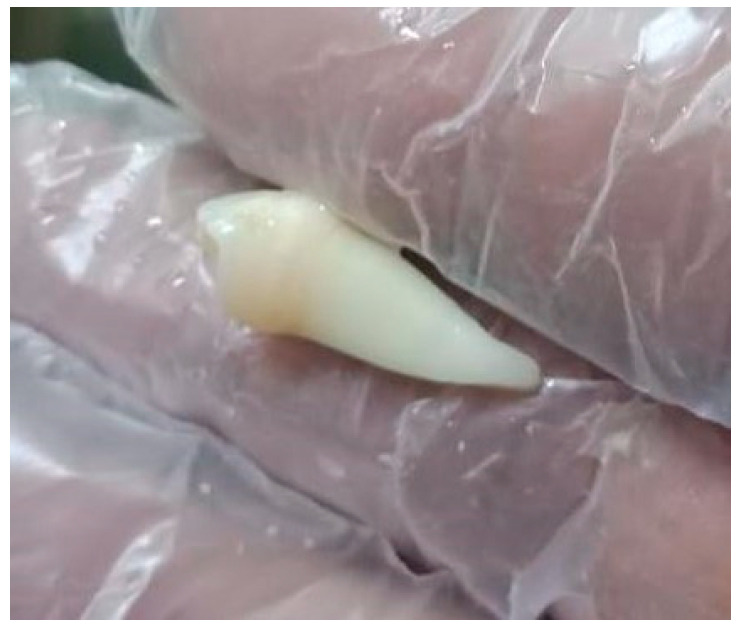
Optical image of one sample of the extracted teeth used in this study.

The research design comprised 27 extracted human teeth, which were obtained in accordance with the Declaration of Helsinki’s guidelines and ethical protocol. The protocol of the study was approved by the Faculty of Dentistry Beni-Suef University Research Ethics Committee (FDBSU-REC, approval number REC-FDBSU/02032023-04/KH). Figure 1 shows a photograph of a sample of the teeth used in this study.

The samples were divided into three groups, where group A samples were subjected to a *Moringa oleifera* extract, and group B were subjected to bioactive glass nanoparticles. Group C was kept as the control group, to be subjected only to the femtosecond laser light. There were *n* = 9 samples for each group A, B, and C.

The materials used in this investigation were applied in accordance with the manufacturer’s instructions; a list of them is provided in Table 1.

### 2.2. Methodology

#### 2.2.1. Sample Preparations

The samples were immersed in 2.6% sodium hypochlorite for seven days to disinfect them. The samples were prepared to mimic stage 3 dentin decay by drilling into each sample at 3 mm diameter and 4 mm depths till leathery dentin texture using a MEISINGER^®^ Model Fissure Carbide Bur size 012 (Meisinger DE, Neuss, Germany), Germany mounted on the High-Speed Handpiece (Sirona T3 Racer, with a maximum rpm of 400,000, Germany).

**Figure 2:**
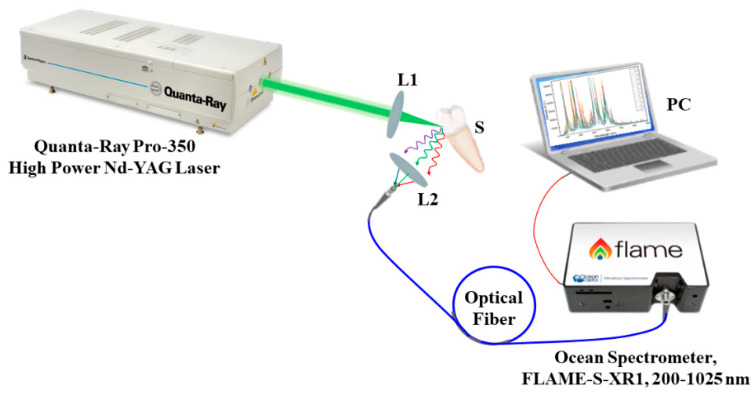
Schematic diagram of the LIBS experimental setup. L1 and L2 are convex lenses; S is the sample.

Thereafter, the elemental analysis of all samples was conducted using the LIBS, which allowed depth profiling and was used to assess the demineralized dentin layer for all groups in this context [20,21]. The Quanta-Ray PRO 350 s-harmonic Nd:YAG laser system from Spectra Physics (Spectra-Physics Inc., Milpitas, CA, USA), with a pulse width of 10 ns and a repetition rate of 10 Hz, constituted the LIBS experimental setup as illustrated in Figure 2. A Gaussian distribution with the TEM00 spatial mode, the highest pulse energy of 1400 mJ, and pulse-to-pulse stability for more than 99% of pulses composed the laser beam’s spatial profile. By adjusting the discharge voltage of the laser flashlamps, the pulse energy was regulated. A convex lens with a 100 mm focal length focused the laser beam on the sample’s surface with shot-to-shot aiming stability of less than ±50. To ensure exact placement control, the sample was mounted on three translation stages. The 100 μm diameter laser point had a pulse energy of roughly 20.5 mJ. The plasma emission was collected using a VIS collimating lens (74-VIS) and steered to the Ocean Spectrometer (Ocean Optics, Inc., Orlando, FL 32817, USA) via an optical premium fiber (QP600-2-SR) (FLAME-S-XR1) (Ocean Optics, Inc., Orlando, FL 32817, USA). A delay time of 1 μs and an integration time of 10 μs were used to acquire the LIBS spectra. Atmospheric pressure was used for all exterior radiations.

Following that, the samples of groups A, B, and C were split into three subgroups, such that each subgroup was activated with the femtosecond laser light at a different wavelength. The subgroups and their respective abbreviations are presented in Table 2.

All group A samples were cavity filled with an even paste of the *Moringa oleifera* plant, and all group B samples were cavity filled with the bioactive glass nanoparticles. Group C was kept as the control group in which no antimicrobial agent was applied to the samples.

#### 2.2.2. Activation of Teeth with Femtosecond Laser Light

The Spectra-Physics INSPIRE HF100 laser system (Spectra-Physics Inc., Milpitas, CA, USA), which was pumped by a mode-locked Ti:sapphire Mai-Tai HP (Spectra-Physics Inc., Milpitas, CA, USA), was used for the photoactivation [22], having a wavelength range of 690 to 1040 nm, an average laser beam strength of 1.5–2.9 W, an 80 MHz repetition rate, and a 100 fs pulse duration. A Newport 843R power meter (RK Tech LTD, Budapest, Hungary) was utilized to measure the knife-edge laser beam waist and track the laser beam strength.

**Figure 3:**
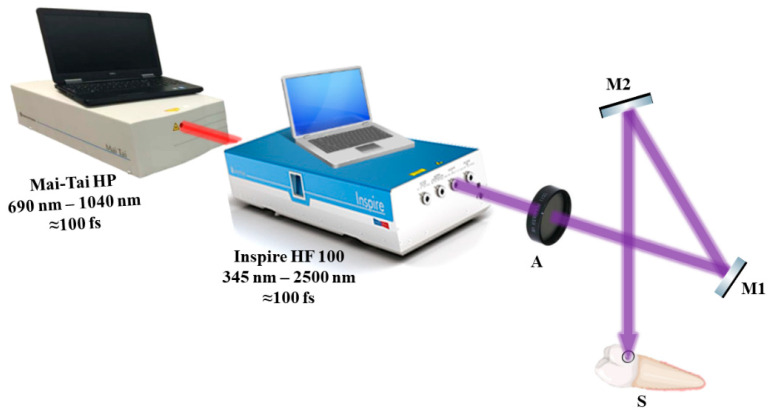
The photoactivation experimental setup showing the method of sample exposure to femtosecond laser light. A—attenuator; M1 and M2—highly reflecting mirrors; S—sample.

Figure 3 depicts the setup for exposing teeth to a laser beam. The laser beam was directed to the samples using highly reflective mirrors M1 and M2, and the laser beam attenuator A was used to regulate the laser beam’s strength. To standardize and enable better sample location at the irradiation setup, each sample was implanted in a petri dish and placed between a light beam, a collecting camera surface, and the tip of the unit.

A pilot study was conducted to determine the ideal parameters for the investigation and the wavelengths that would not distort the samples. Three specific wavelengths were selected to encompass the three electromagnetic spectrum ranges of ultraviolet (390 nm), visible (445 nm), and near-infrared (780 nm), which are all covered by the femtosecond laser light wavelengths. Accordingly, to prevent overstimulation, the beam was concentrated and aimed toward the agents (*Moringa oleifera* and bioactive glass nanoparticles) that had been applied to the prepared samples’ dentin surfaces. The dentin surfaces were exposed to a perpendicular laser beam that was positioned roughly 10 cm above each sample during the irradiation processes. To start the remineralization activity of the dentin surface, the power of the femtosecond laser was adjusted to milliwatts (300 mW). The photoactivation duration was set at 5 min [23].

After the femtosecond laser light activation, the samples were sealed with a temporary filling (BMS DENTAL (BMS Dental, Capannoli, PI, Italy); see Table 1) and then submerged in a sample divider container with distilled water for 68 days.

The samples of groups A and B followed the same methodology, with the agent applied on the samples being the difference. The samples of group C followed the same methodology, noting the difference that in this group, no antimicrobial agent was applied.

#### 2.2.3. Elemental Analysis Using LIBS

For all samples in groups A and B the temporary filling and the applied antimicrobial agent were manually removed, rinsed with distilled water, and dried with cotton.

For all subgroups at the three different wavelengths, to observe the photoactivation elemental change results in real time, the LIBS was used as an indicator and reporter due to its specificity and sensitivity as it reports the qualitative and quantitative analysis for the samples used by photoactivation in the content with the LIBS spectrum. The samples were implanted longitudinally to allow homogeneous and repeatable readings under the tissue surface. The laser intensity being initially perpendicular to the dentin sample’s long axis resulted in plasma formation. With a set pulse duration of 10 ns and a pulse repetition rate of 10 Hz, dentin tissues were subjected to radiation at an energy per pulse of about 20.5 mJ [24]. The surface profile was then monitored by the spectrometer to track the changes in the mineral content of the dentin using laser light at a wavelength of 532 nm LIBS surface profile.

## 3. Results and Discussion

**Figure 4:**
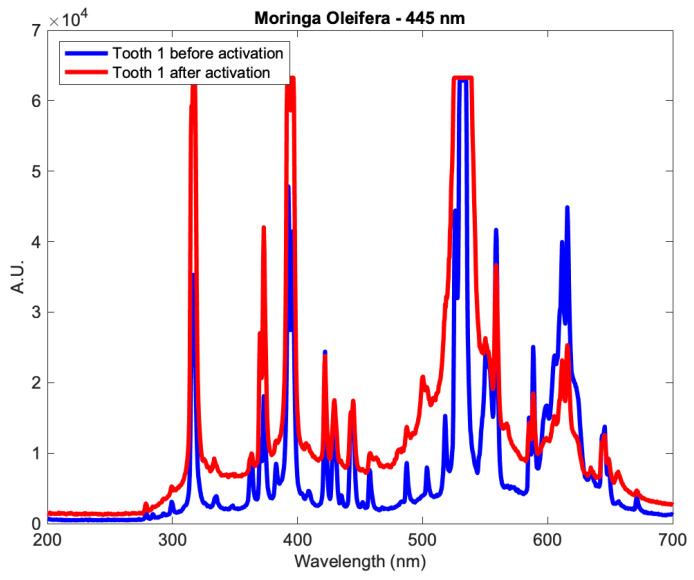
The profile of a tooth from subgroup Aλ_2_ before and after applying the *Moringa oleifera* extract and photoactivation at 445 nm.

The results of the real-time elemental analysis of the mineral content on the dentin surface for all groups were gathered using LIBS. The LIBS surface profiles of the samples were measured in absolute units (A.U.) and presented as a function of wavelength, in which different wavelengths corresponded to different minerals. An example of the LIBS surface profiles is shown in Figure 4, for a tooth from subgroup Aλ_2_ as it showed significant change before and after activation.

The database of the National Institute of Standards and Technology of the US Department of Commerce (NIST) was used to obtain a profile of the reference mineral content at a specific wavelength before applying the antimicrobial agent and before photoactivation [25]. The elements relevant to the minerals in dental tissue were selected, where each element appears at a specific wavelength in the electromagnetic spectrum. Specifically, Ca I appears at 422 nm, Ca II appears at a range of wavelengths 393–396 nm, P appears at 213 and 253 nm, O appears at 395, 436, 496 and 533 nm, C appears at 247 nm, Mg I appears at 285 nm, Mg II appears at 280 nm, Na appears at 590 nm, and F II appears at 385 and 402 nm.

For comparison purposes, the change in mineral content at specified wavelengths was measured as a relative change in percentage, by computing the percentage change in the mineral content before and after, relative to before. The relative change was computed as

(1) $$Relative\;change\;\left(\lambda ,\;x_{\lambda },\;x_{ref,\lambda }\right)=\frac{x_{\lambda }-x_{ref,\lambda }}{x_{ref,\lambda }}\times 100\%$$

where *λ* is the wavelength, *x_ref,λ_* is the reference mineral content at a specific wavelength before photoactivation, and *x_λ_* is the mineral content at a specific wavelength after photoactivation.

**Figure 5:**
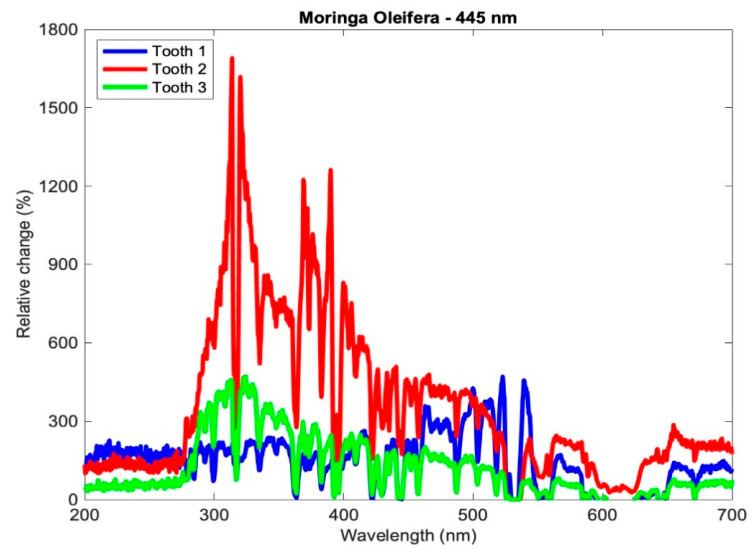
Relative change in the mineral content of the samples of subgroup Aλ_2_.

Therefore, a positive relative change indicates an increase in the content of the mineral at the indicated wavelength, and vice versa. The relative changes of the teeth of subgroup Aλ_2_ are shown in Figure 5.

**Figure 6:**
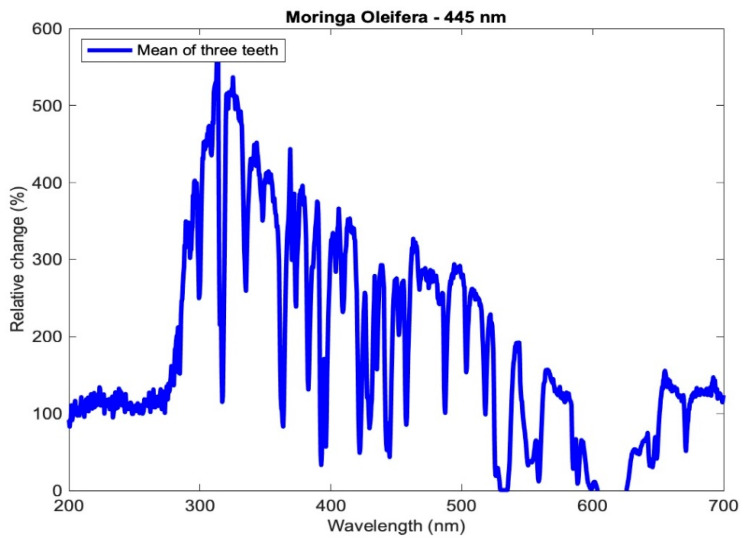
Relative change in the mineral content of the aggregated profiles of subgroup Aλ_2_.

For comparison purposes between the different subgroups, each subgroup was aggregated, meaning that the mineral contents of the samples of each subgroup were averaged at each wavelength, once before applying the agents and photoactivation and once after. Afterward, the relative change in the elemental content was computed for the aggregated profiles. The mean relative change was computed as

(2) $$Mean\;relative\;change\;\left(\lambda ,\;{\bar{x}}_{\lambda },\;{\bar{x}}_{ref,\lambda }\right)=\frac{{\bar{x}}_{\lambda }-{\bar{x}}_{ref,\lambda }}{{\bar{x}}_{ref,\lambda }}\times 100\%$$

where ${\bar{x}}_{ref,\lambda }$ is the average of the reference mineral content at a specific wavelength before the photoactivation of the three teeth of each subgroup, and ${\bar{x}}_{\lambda }$ is the average mineral content at a specific wavelength after the photoactivation of the three teeth of each subgroup. The relative change of the averaged profiles for subgroup Aλ2 is shown in Figure 6.

**Figure 7:**
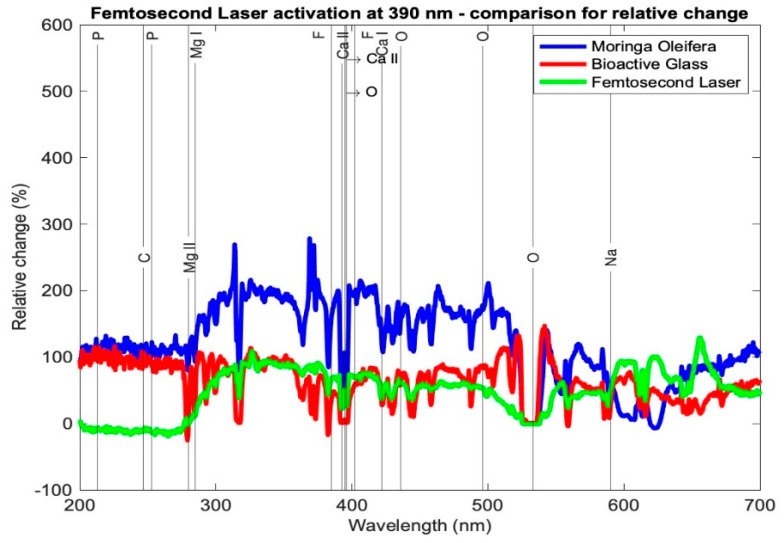
Relative change profile for subgroups Aλ_1_, Bλ_1_, and Cλ_1_. The elements of interest in dentin remineralization are also shown in the figure.

**Figure 8:**
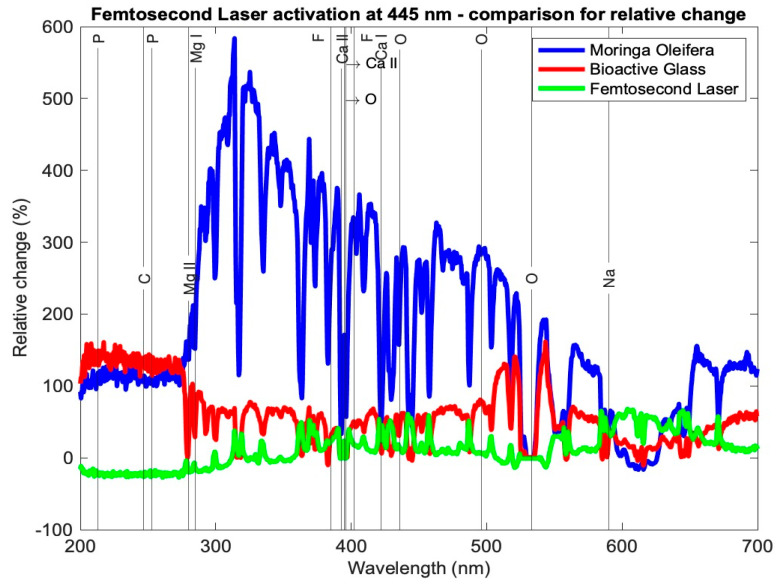
Relative change profile for subgroups Aλ_2_, Bλ_2_, and Cλ_2_. The elements of interest in dentin remineralization are also shown in the figure.

**Figure 9:**
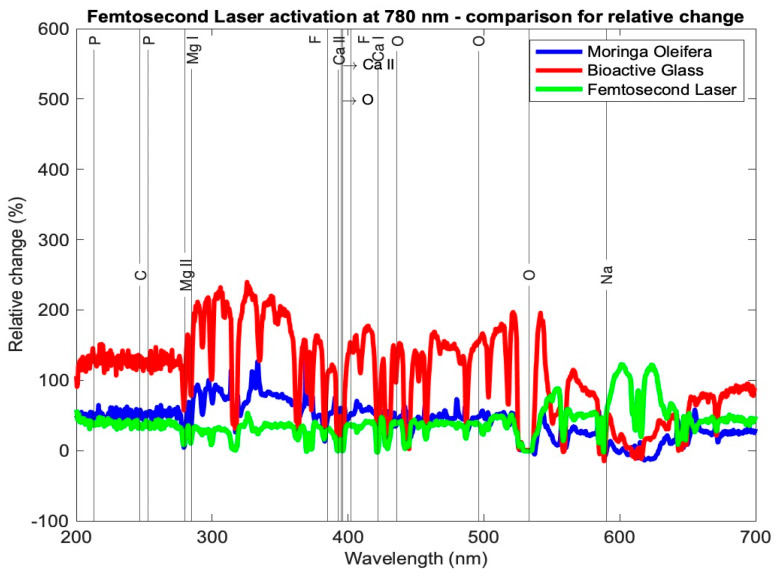
Relative change profile for subgroups Aλ_3_, Bλ_3_, and Cλ_3_. The elements of interest in dentin remineralization are also shown in the figure.

Table 3 presents the relative change in each mineral content for the average profile of each subgroup for the tested samples using the three setups: *Moringa oleifera* photoactivation with femtosecond laser light at three different wavelengths, bioactive glass nanoparticles photoactivation with femtosecond laser light at three different wavelengths, and photoactivation femtosecond laser light only at three different wavelengths. In general, all elements exhibited a relative increase, other than a few elements in subgroup Cλ_2_ where there was a relative decrease. Figure 7, Figure 8 and Figure 9 present the relative change profiles for subgroups (Aλ_1_, Bλ_1_, Cλ_1_), (Aλ_2_, Bλ_2_, Cλ_2_), and (Aλ_3_, Bλ_3_, Cλ_3_), respectively. The elements of value for the remineralization are also presented in the figures.

The results presented Table 3 and Figure 7, Figure 8 and Figure 9 demonstrate the relative change of each mineral content separately from the average tested sample from each subgroup. Table 3 shows the relative changes in the nine research subgroups, with the first column listing the significant elements found in tooth tissue and the second column listing each element’s wavelength in the electromagnetic spectrum according to the NIST data source. As an illustration, subgroup Aλ_1_ represents the mean of the samples in this group and the percentage change in each mineral, signifying that the Ca I element grew 120%, the Mg I element increased 117%, and the F II element at 402 nm increased 197% after treatment.

In Table 3, the highest increase in element content is shaded in green color. It is evident that Ca I for subgroup Aλ_1_ showed the highest relative change at 120%; Ca II at 393 and 395 nm had the highest relative changes in subgroup Aλ_2_ at 48% and 67%, respectively. The element P showed the highest relative change from subgroups Cλ_3_ and Bλ_2_, where the profiles at 213 and 253 nm showed increases of 149% and 123%, respectively. Elements Mg I and Mg II exhibited the largest relative changes from subgroup Aλ_2_ at 189% and 136%, respectively. Finally, element F II at 385 and 402 nm had the highest relative increases in the subgroup Aλ_2_ at 271% and 328%, respectively.

Hydroxyapatite is the major component required for the biomimetic remineralization of dentin, in which the main elements to observe as indicators of remineralization are Ca, P, Mg, and F. Significant changes in the mineral content of all samples show that biomimetic remineralization occurred in the mineral content of all tested setups. However, the changes were not the same.

Looking at the overall picture, the obtained results presented in Table 3 suggest that subgroup Aλ_2_, with *Moringa oleifera* extract acting as an agent combined with femtosecond laser light at 445 nm, resulted in the highest change in content for most of the observed elements. It had the largest positive relative change for elements Ca II, O, Mg I, Mg II, and F II and the second highest positive change in Ca I. For element P, although not the highest relative to the other subgroups, it had a high increase in content, with a value larger than 100%. Finally, for elements C and Na, the relative changes were positive and quite high when compared with the other subgroups, being 115% and 45%, respectively, where their ranges of relative change from all subgroups ranged from −27 to 135% and −3 to 51%, respectively.

Studies in the literature have shown that diode laser treatment at 445 nm prevents enamel demineralization and cavity disinfection [26,27,28]. The results of this study agree that 445 nm is the best wavelength, and the results presented herein show that using a femtosecond laser with *Moringa oleifera* not only prevents demineralization but in fact causes remineralization.

The second overall increases in content were achieved by the *Moringa oleifera* extract photoactivated with the femtosecond laser at 390 nm (subgroup Aλ_1_), whereas the third best overall increases in content were achieved by bioactive glass nanoparticles photoactivated with the femtosecond laser at 780 nm (subgroup Bλ_3_).

For the group C, the control group, even though the femtosecond laser light showed that the best results were achieved at a wavelength of 390 nm (subgroup Cλ_1_) and were not as high as those of the other subgroups of the same wavelength, it is important to highlight these results were achieved without any antimicrobial agents added to the samples, and the mineral content increased with femtosecond laser light only.

A comparison between the other subgroups showed that Aλ_3_, Bλ_1_, Bλ_2_, Cλ_2_, and Cλ_3_ exhibited the smallest increases in the element content for most of the samples. It is worth noting that for the element Ca I, the largest relative increases occurred in subgroup Aλ_1_ at 120%, and this change was almost double the second highest change in this element from the eight other subgroups.

Within the limitations of this study, the observed change of mineral content allows this study to be used as a reference to enhance and improve dental treatment following the conservative modern medicine by using noninvasive treatment for the samples. It may be concluded that the femtosecond laser light photoactivation clearly enhanced the biomimetic remineralization, particularly at 445 nm using *Moringa oleifera* as an antimicrobial enhancing agent.

The findings of this study agree with findings in the literature. For example, bioactive glass nanoparticles, due to their biosafety, have been employed extensively in enamel remineralization but less commonly in dentin remineralization [29,30,31]. The results presented herein agree that bioactive glass nanoparticles, although they did change the mineral content, were not the superior choice.

It is also supported in the literature that the two indicators calcium serum and phosphorus serum may be improved by *Moringa oleifera* leaf extract [32]. The findings herein support this, and we further photoactivated them by femtosecond laser light. The superiority of combining both the laser and the extract was evident at 445 nm, as opposed to the control group, which was photoactivated only without the extract.

The findings that photoactivation using a femtosecond laser only at 390 nm exhibited a significant change in mineral content, specifically, phosphorus, may open up a new method for the biomimetic remineralization of dentin, keeping in mind that phosphorus is essential for normal development, healing, and regeneration [33].

## 4. Further Investigations

This preliminary study opens up areas of further research. For example, one area could be to first photoactivate with the femtosecond laser light at 390 nm and follow that by applying the *Moringa oleifera* extract and photoactivating it at 445 nm to study if that increases the biomimetic remineralization.

Another study to be carried out may be a time sensitivity analysis of the applied antimicrobial agents. Does the amount of time the agent is applied on the tooth matter? If so, what is the optimum time that an agent should be applied for?

Another further investigation may be to confirm whether the remineralization results obtained in this study are better than those of gold standard techniques or not.

## 5. Conclusions

For enhancing biomimetic dentin remineralization in stage 3 dentin decay treatment, where tooth decay has reached the dentin layer, femtosecond laser photoactivation with or without antimicrobial agents fulfills the aim. The degree of remineralization depends on a set of variables, including the activation wavelength and the applied agent, if any. Of the tested experiments, the best results were achieved with *Moringa oleifera* when photoactivated with femtosecond laser light at 445 nm.

Topics involving dentin remineralization with lasers are still novel and subjects to further investigate. The dentin remineralization response from our study can be used for further investigations with different settings, materials, pulse durations, and wavelengths. Additionally, our method suggests that the biomimetic dentin remineralization with femtosecond photoactivation is quite promising by obtaining the optimal wavelength with power and duration from the information collected. More applications and research will be conducted utilizing this strategy as a result of our demonstration proving the biomimetic remineralization impact.

Our analysis also proves that the LIBS is helpful in the field of biomimetic remineralization of materials since it is a real-time, dependable analytical method that can be used to monitor elemental changes in a clinical context without deforming the sample. To our knowledge, the use of LIBS in analyzing dental tissue is only used in scientific purposes and has not been considered as a clinical setting tool. The findings of this study may support their use in clinical settings; if the LIBS device evolves into a handheld detection instrument that can indicate and validate the continuity of the treatment, the same sample might be directly treated to it.

## Figures and Tables

**Table 1:** List of obtained materials, their manufacturer, and their texture.

Material	Manufacturer	Texture
*Moringa oleifera*	Egyptian Scientific Society of Moringa, National Research Centre, Giza, Egypt	Moringa leaf extracts
Bioactive glass (Batch #: NT-BG45S5)	NanoTech Egypt, Giza, Egypt	White, powder, average size 50 ± 20 nm, spherical-like shape
Temporary filling	BMS DENTAL, Italy	White, paste

**Table 2:** The groups and subgroups of teeth samples used in the experiments in this study.

Group	Antimicrobial Agent	Sample Size	Activation Wavelength (nm)	Subgroup Abbreviation
A	*Moringa oleifera*	3	390	Aλ_1_
		3	445	Aλ_2_
		3	780	Aλ_3_
B	Bioactive glass nanoparticles	3	390	Bλ_1_
		3	445	Bλ_2_
		3	780	Bλ_3_
C	None	3	390	Cλ_1_
		3	445	Cλ_2_
		3	780	Cλ_3_

**Table 3:** Relative change in relevant element content for the nine subgroups of this study.

		Relative Change in Subgroups, Measured in %								
Element	λ (nm)	Aλ_1_	Bλ_1_	Cλ_1_	Aλ_2_	Bλ_2_	Cλ_2_	Aλ_3_	Bλ_3_	Cλ_3_
Ca I	422	120	28	37	49	10	24	40	4	−1
Ca II	393	47	1	24	48	1	−1	12	12	−1
Ca II	396	52	1	25	67	1	−1	13	10	0
P	213	112	93	−6	119	149	−21	47	130	35
P	253	123	86	−14	103	114	−19	50	122	33
O	394	99	5	46	171	6	14	56	39	11
O	436	160	62	60	225	47	14	40	126	40
O	496	178	85	53	286	79	7	49	154	38
O	533	1	1	−1	1	1	−1	1	1	−1
C	247	117	107	−11	115	132	−27	58	135	33
Mg I	285	117	45	12	189	51	−14	48	117	22
Mg II	280	94	11	0	136	25	−20	17	89	19
Na	590	50	26	49	45	27	35	−3	31	51
F II	385	163	38	68	271	30	24	47	111	30
F II	402	197	58	68	328	51	18	53	150	35

## Data Availability

Not applicable.
